# The MMP-1/PAR-1 Axis Enhances Proliferation and Neuronal Differentiation of Adult Hippocampal Neural Progenitor Cells

**DOI:** 10.1155/2015/646595

**Published:** 2015-12-13

**Authors:** Maria Maddalena Valente, Megan Allen, Valeria Bortolotto, Seung T. Lim, Katherine Conant, Mariagrazia Grilli

**Affiliations:** ^1^Laboratory of Neuroplasticity, Department of Pharmaceutical Sciences, University of Piemonte Orientale “Amedeo Avogadro”, 28100 Novara, Italy; ^2^Department of Neuroscience, Georgetown University Medical Center, Washington, DC 20057, USA

## Abstract

Matrix metalloproteinases (MMPs) are zinc-dependent endopeptidases that play a role in varied forms of developmental and postnatal neuroplasticity. MMP substrates include protease-activated receptor-1 (PAR-1), a G-protein coupled receptor expressed in hippocampus. We examined proliferation and differentiation of adult neural progenitor cells (aNPCs) from hippocampi of mice that overexpress the potent PAR-1 agonist MMP-1. We found that, as compared to aNPCs from littermate controls, MMP-1 tg aNPCs display enhanced proliferation. Under differentiating conditions, these cells give rise to a higher percentage of MAP-2^+^ neurons and a reduced number of oligodendrocyte precursors, and no change in the number of astrocytes. The fact that these results are MMP and PAR-1 dependent is supported by studies with distinct antagonists. Moreover, JSH-23, an inhibitor of NF-*κ*B p65 nuclear translocation, counteracted both the proliferation and differentiation changes seen in MMP-1 tg-derived NPCs. In complementary studies, we found that the percentage of Sox2^+^ undifferentiated progenitor cells is increased in hippocampi of MMP-1 tg animals, compared to wt mice. Together, these results add to a growing body of data suggesting that MMPs are effectors of hippocampal neuroplasticity in the adult CNS and that the MMP-1/PAR-1 axis may play a role in neurogenesis following physiological and/or pathological stimuli.

## 1. Introduction

Newly born neurons that are generated in the dentate gyrus (DG) of the adult hippocampus are increasingly appreciated as potential effectors of cognitive flexibility, pattern separation, and spatially precise search strategies [1–3]. Dysregulated adult hippocampal neurogenesis (AHN) is a correlate of varied physiological and pathological states, and may contribute to and/or result from the same. While adult neurogenesis can be modulated by numerous stimuli, several conditions characterized by an increase in progenitor cell proliferation are coincidently associated with an increase in the level and activity of secreted enzymes belonging to the matrix metalloproteinase (MMP) family [4–6]. Prior studies have also implicated MMP activity in the proliferation of varied cell types including neural progenitors [7], but the overall role of specific MMPs in aNPC proliferation and the mechanism(s) or substrates important to MMP-dependent effects are not particularly well understood.

Potential protease substrates that are highly expressed within the adult hippocampus include synaptic cell adhesion molecules and protease-activated receptor-1 (PAR-1), a GPCR activated by cleavage of N-terminal sequence which in turn exposes a tethered peptide ligand [8–10]. PAR-1 is of particular interest to neurogenesis since it is expressed in the DG and found on both neurons and glial cells [8, 11]. Moreover, its signaling has been linked to increases in the function of NMDARs thought to play a role in neurogenesis as well as to increased release of soluble factors that enhance progenitor cell proliferation [8, 12, 13].

PAR-1 activators include thrombin and APC, two blood-derived proteases that may enter the CNS in response to blood brain barrier damage as well as a small subset of MMPs (MMP-1 and MMP-13) [9, 14, 15]. MMP-1 in particular is equipotent to thrombin in its ability to stimulate receptor cleavage and PAR-1 dependent intracellular signaling [14]. Of note, however, is the fact that MMP-1 cleavage of PAR-1 generates a relatively unique tethered peptide ligand since cleavage occurs as a site that is two amino acids N-terminal to the thrombin cleavage site [15].

MMP-1 has the potential to be an important PAR-1 agonist in the absence of blood brain barrier damage. It is expressed by astrocytes [16], and while basal expression is typically low, levels of MMP-1 may be increased in association with physiological and pathological stimuli that are linked to neurogenesis [17–19]. MMP-1 dependent activation of PAR-1 has, however, not been well explored with respect to its function in central nervous system physiology or neurogenesis in particular.

Quiescent neural stem/progenitor cells, thought to generate neurons that will ultimately mature, express markers including GFAP [20]. In the present study, we therefore take advantage of a unique mouse model in which human MMP-1 is expressed under the control of a GFAP promoter to examine the MMP-1/PAR-1 axis in adult hippocampal neurogenesis. Human MMP-1 is an orthologue of the much later identified murine MMP-1a [21], and, importantly, it activates murine PAR-1 [22]. We show that the MMP-1/PAR-1 axis can increase both progenitor cell proliferation and neuronal fate specification and we suggest that future studies are warranted to determine if this axis is important to progenitor cell plasticity in physiological settings such as exercise and environmental enrichment as well as in pathological conditions.

## 2. Materials and Methods

### 2.1. Animals

Adult male (3-4-month-old) wild type C57/BL6 or heterozygous littermate MMP-1 tg mice on a C57/BL6 background were used in the present study. The MMP-1 tg animals were prepared at the Johns Hopkins University via pronuclear injection of a glial fibrillary acid protein- (GFAP-) MMP-1 construct. This construct encodes human MMP-1, an orthologue of the much later identified mouse MMP-1a [21]. Importantly, human MMP-1 cleaves murine PAR-1 [22]. We obtained the MMP-1 construct from Dr. Jeanine D'Armiento who has used it to overexpress MMP-1 in mouse's heart and lung [23, 24]. The generation of the combined GFAP-MMP-1 construct was carried out using a GFAP promoter from Dr. Michael Brenner [25]. All experimental procedures were performed with approval of the Johns Hopkins University and Georgetown University Animal Care and Use Committee guidelines.

### 2.2. Isolation and Culture of Adult Neural Progenitor Cells (aNPCs) from Murine Hippocampus

For each aNPC preparation, three adult (3-4-month-old) male mice were humanely euthanized. The brains were extracted and hippocampi were isolated under a dissecting microscope using fine surgical instruments and collected in ice-cold PIPES buffer pH 7.4 containing 20 mM PIPES, 25 mM glucose, 0.5 M KCl, 0.12 M NaCl (Sigma, St. Louis, MO), and 100 U/100 *μ*g/mL Penicillin/Streptomycin solution (Invitrogen, Carlsbad, CA). After centrifugation (110 ×g × 5 min), tissue was digested for 40 minutes at 37°C using the Papain Dissociation System (Worthington DBA, Lakewood, NJ). The cell suspension was placed into 25 cm^2^ Falcon cell-culture flasks (Thermo Fisher Scientific, Rockville, MD) and cultured in growth medium [Neurobasal-A medium containing B27 supplement, 2 mM L-glutamine (Invitrogen), 10 ng/mL human basic fibroblast growth factor (bFGF-2, PeproTech, Rocky Hill, NJ), 20 ng/mL human epidermal growth factor (EGF, Sigma), 4 *μ*g/mL heparin sodium salt (Sigma), and 100 U/100 *μ*g/mL Penicillin/Streptomycin]. Primary (Passage 1, P1) neurospheres were passaged after 7–9 days* in vitro* (DIV), whereas subsequent passages were prepared every 5 DIV. At each passage, cells were plated in a T25 flask at a density of 12,000 cells/cm^2^ in growth medium. P3–P10 neurospheres were utilized for experiments.

### 2.3. aNPC Differentiation and Proliferation

For evaluation of cell proliferation, dissociated aNPCs were plated onto LuminNUNC F96 MicroWell plates (Thermo Fisher Scientific), at a density of 4,000 cells per well, in growth medium in presence of 1 *μ*M BMS-200261, 8 *μ*M MMP inhibitor II, 3 *μ*M JSH-23 (Calbiochem, Merck KGaA, Darmstadt, Germany), or vehicle for 0 to 72 h, as indicated. Proliferation rates were determined by using the CellTiter-Glo Luminescent Cell Viability Assay (Promega, Madison, WI), according to the manufacturer's instructions. All experiments were performed in triplicate, and data (expressed as counts per second) represent mean ± standard deviation (SD), as indicated.

For differentiation experiments, we used a previously described protocol [26]. Briefly, neurospheres were dissociated and plated onto laminin-coated chamber slides in Neurobasal-A medium with B27 supplement and 2 mM L-glutamine. At plating, NPCs were treated with vehicle, 1 *μ*M of the PAR-1 antagonist BMS-200261 trifluoroacetate (Sigma), or 3 *μ*M JSH-23 (Calbiochem). After 24 h, cells were washed in PBS and fixed with ice-cold 4% PFA for 20 min at RT for subsequent immunofluorescence analysis. In each experiment, 5 fields/well (corresponding to 100–150 cells/well) were evaluated. All experiments were run in triplicate, and experiments were repeated at least three times. Data represent the mean value ± SD as noted. In parallel, the survival rate in culture was evaluated in each fixed culture as previously described [26].

### 2.4. Immunocytochemical Analysis

After fixation, neurosphere-derived differentiated cells were washed three times in PBS and permeabilized in PBS containing 0.48% (vol/vol) Triton X-100 (Sigma) for 5 min at RT. The primary antibodies against MAP-2 (rabbit polyclonal, 1 : 600, Chemicon, Temecula, CA), GFAP (mouse monoclonal, 1 : 600, Millipore, Billerica, MA), or NG2 (rabbit polyclonal, 1 : 500, Abcam, Cambridge, MA) were incubated for 150 min at RT in an antibody solution containing 16% (vol/vol) goat serum. Secondary antibodies were as follows: Alexa Fluor 488 conjugated goat anti-rabbit antibody(1 : 1600, Invitrogen) and Alexa Fluor 488 conjugated goat anti-mouse antibody (1 : 1600, Invitrogen). Nuclei were counterstained with DAPI (1 : 10000, Sigma). Fluorescent Mounting Medium (Electron Microscopy Sciences, Hatfield, PA) was applied to slides as antifading agent prior to addition of coverslips.

### 2.5. Protein Isolation and Western Blot Analysis

For protein isolation, aNPC were lysed in 3x vol/vol of ice-cold hypotonic RIPA buffer [50 mM Tris-HCl pH 7.5, 150 mM NaCl, 0.5 mM EDTA pH 8, 1% (vol/vol) Triton X-100, 0.1% (wt/vol) SDS, 1 mM DTT, protease inhibitor mix, and phosphatase inhibitor cocktails 2 and 3 (Sigma)] for 25 min on ice. Lysates were centrifuged at 1,300 ×g for 10 min at 4°C and supernatants were collected. Protein concentration was determined by Pierce BCA protein assay kit (Pierce, Rockford, IL) and equivalent amounts of each sample were separated onto SDS-PAGE gel and transferred onto nitrocellulose membranes. Molecular weights were inferred by comparison to prestained markers (BioRad, Hercules, CA). Membranes were then blocked in 5% nonfat dry milk in phosphate-buffered saline (PBS) with 0.1% Tween (PBST) for 1 h. The blot was subsequently probed with a primary antibody to PAR-1 (H-111, 1 : 500, Santa Cruz Biotechnology, Santa Cruz, CA). After washing the membrane in PBST, it was incubated with an appropriate secondary antibody. The membrane was then washed again in PBST and immunoreactive bands were visualized using electrochemiluminescence (Amersham Pharmacia Biotech, Piscataway, NJ, USA).

### 2.6. ELISA

ELISA for total MMP-1 was performed on neurosphere supernatants using a commercially available kit (R&D Systems, Minneapolis, MN) according to the manufacturer's instructions.

### 2.7. Flow Cytometry

Animals were euthanized with CO_2_ intoxication and immediately perfused with PBS. For each experiment, two hippocampi from littermates of the same genotype (WT, *n* = 2; hMMP-1 tg, *n* = 2) were microdissected and combined in a 15 mL conical tube filled with 10 mL of ice-cold dissection medium (Ca^2+^ and Mg^+^ free HBSS, 0.01% 100 mM Sodium Pyruvate, 0.10% Glucose, and 0.01% 1 M HEPES) and gently washed 2 times. Dissection medium was aspirated and tissues were incubated in 5 mL of 0.25% Trypsin in HBSS plus phenol red (Invitrogen) at 37°C for 10 minutes. Next, 0.5 mL of 1% DNase solution was added to tube and incubated on bench for 3 minutes. This volume was aspirated and tissue was gently washed 3 times in temperature equilibrated plating medium (MEM with Earle's BSS, 10% FBS, 0.45% glucose, 0.01% 100 mM Sodium Pyruvate, 0.01% 200 mM Glutamine, and 0.01 Penicillin/Streptomycin). Tissues were then triturated in 2 mL total volume of plating medium followed by filtration through a 70 *μ*m pore. Cell suspension was gently centrifuged and supernatant discarded. Next, cells were resuspended in 4% paraformaldehyde/PBS for 15 min with gentle rotation at room temperature. After one wash and centrifugation in PBS, cell membranes were permeabilized in 100% MeOH for 10 min at 4°C. Cells were washed 3 times in PBS and incubated overnight in antibody solution (Phycoerythrin (PE) conjugated anti-SOX2; 656103, BioLegend, San Diego, CA) made up in PBS-TX at 4°C. An aliquot of cells was used as autofluorescence control. Incubations were performed at 4°C overnight. Flow cytometric analysis was performed with a BD FACS Aria III machine and FCS express software by De Novo. For each of the gated populations the percentage and geometric mean fluorescence intensity (MFI) were analyzed.

### 2.8. Statistical Analysis

For all experiments using* ex vivo* aNPCs data are expressed as mean ± SD or SE, as indicated, of at least three experiments in triplicate. Data were analyzed by two-tailed, unpaired, Student's *t*-test for comparison of two groups or ANOVA, followed by Tukey's post hoc analysis, for comparison of groups greater than two. Statistical significance level was set for *p* values less than or equal to 0.05.

## 3. Results

### 3.1. aNPCs from MMP-1 tg Animals Show Increased Proliferation

**Figure 1:**
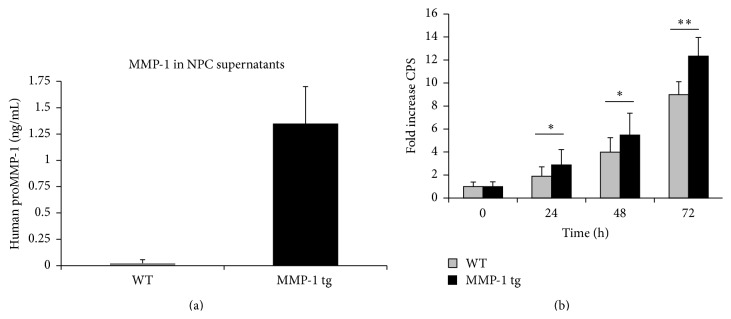
aNPCs from MMP-1 tg animals release MMP-1 and have an increased proliferation rate. (a) Supernatants from tg cultures express hMMP-1 as detected by ELISA. (b) Time course analysis of proliferation for WT and MMP-1 tg-derived aNPC. Cells from tg animals display an increased proliferation rate, compared to WT-derived aNPC (b). Data are the mean ± SD from four replicate wells and are representative of an experiment performed with different NPC preparations on three separate occasions (^*∗*^ *p* < 0.05; ^*∗∗*^ *p* < 0.01 versus WT).

NPCs were harvested and cultured from wild type and MMP-1 tg animals. As shown in Figure 1(a), supernatants from tg cultures express hMMP-1. Cell proliferation was subsequently observed as a function of time in culture in WT-derived and tg-derived cells. Interestingly, aNPCs from tg animals showed enhanced proliferation, when compared to WT aNPC, at 24, 48, and 72 h time points (Figure 1(b)). Results shown are mean ± SD from four replicate wells per genotype and are representative of an experiment performed with different NPC preparations on three separate occasions (^*∗*^ *p* < 0.05; ^*∗∗*^ *p* < 0.01). The fold increase from WT control (0 h) is shown (baseline CPS values were 51,881 ± 10,394 for WT and 51,422 ± 10,473 for MMP-1 tg). Consistent with increased proliferation as opposed to survival, as determined by published techniques [26], cell viability in WT and tg cultures did not differ (mean percentage ± SD of viable cells: WT: 88.8 ± 2.3, *n* = 9; MMP-1 tg: 89.2 ± 1.02, Student's *t*-test).

### 3.2. MMP-1 tg-Derived aNPCs Show Increased Differentiation towards the Neuronal Lineage

**Figure 2:**
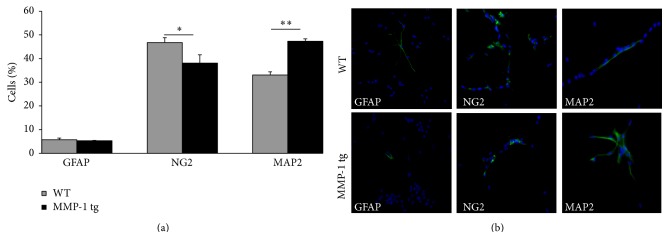
MMP-1 tg-derived aNPCs show increased differentiation towards the neuronal lineage. (a) Differentiation experiments on aNPCs from WT and MMP-1 tg animals. Data, which represent mean ± SD values, are expressed as percentage over total number of viable cells. A statistically significant increased percentage of MAP-2^+^ neurons was generated* in vitro* by aNPCs from hMMP-1 tg, compared to WT counterpart. Conversely, the number of cells expressing the oligodendrocyte precursor marker NG2 is significantly reduced in absence of changes in the number of GFAP^+^ cells in tg cultures (^*∗*^ *p* < 0.05; ^*∗∗*^ *p* < 0.01 versus WT cells, ANOVA). (b) Representative GFAP, NG2, and MAP2 immunostaining (in green) in WT and hMMP-1 tg NPC cultures. Nuclei are counterstained by DAPI (in blue).

To address the question of whether NPC differentiation is altered in the background of enhanced MMP-1 expression, we also performed differentiation experiments on aNPCs from WT and MMP-1 tg animals. Under appropriate conditions (removal of growth factors) NPCs express their multipotentiality and give rise to cells belonging to three neural lineages, namely, neurons, astrocyte, and oligodendrocyte precursors. Results (mean ± SD), as shown in Figure 2(a), suggest that differentiation towards a neuronal lineage is enhanced in the setting of excess hMMP-1. We indeed observed a statistically significant increase in the percentage of MAP-2^+^ cells generated from hMMP-1 tg-derived compared to WT-derived aNPC (^*∗∗*^ *p* < 0.01, Student's *t*-test). Interestingly, this effect was selective on the neuronal fate of MMP-1 tg NPC, since the number of GFAP^+^ astrocytes was not different in the two genotypes, while the percentage of NG2^+^ oligodendrocyte precursors was significantly reduced in tg-derived cultures compared to WT-derived cells (^*∗*^ *p* < 0.05, Student's *t*-test). Of note, there was no genotype effect on the percentage of apoptotic cells in the variously stained preparations (*data not shown*). Representative immunostaining for MAP2, GFAP, and NG2 in WT and hMMP-1 tg cultures is shown in Figure 2(b).

### 3.3. MMP-1 tg aNPC Associated Changes in Proliferation and Differentiation Are PAR-1 Dependent

**Figure 3:**
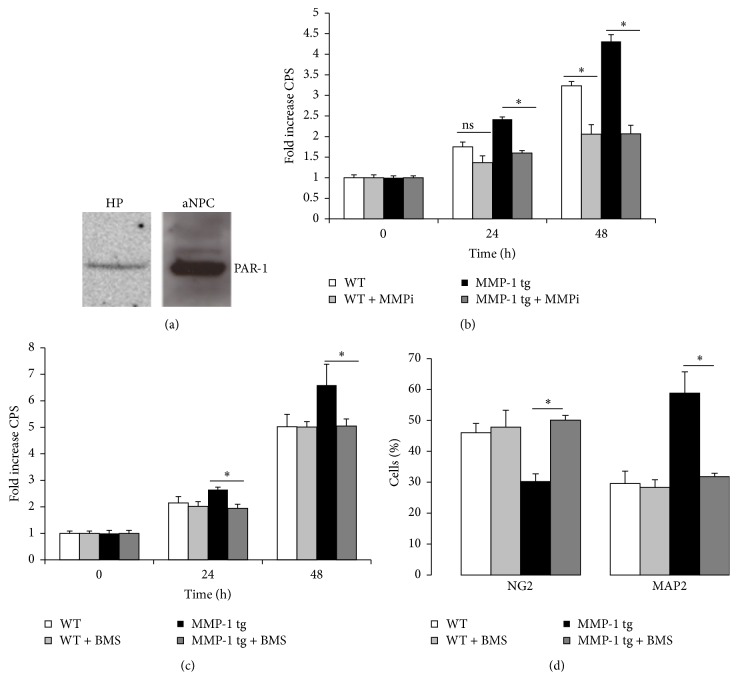
MMP-1 tg-associated changes in aNPC proliferation and differentiation are PAR-1 dependent. (a) Western blot analysis for PAR-1 expression in hippocampal (HP) and WT aNPC lysates. A single band at the expected molecular weight (46 kDa) is detected. (b) Effect of an MMP inhibitor on the proliferation rate of WT and tg cultures. 8 *μ*M MMP inhibitor II (MMPi) significantly reduced aNPC proliferation in tg cultures at 24 h time point and in both WT and tg cultures at 48 h (^*∗*^ *p* < 0.05; ANOVA) (b). (c) Effect of a PAR-1 antagonist on the proliferation of WT and tg cultures. 1 *μ*M BMS-200261 (BMS) reduced proliferation of aNPCs from MMP-1 tg animal. (d) Effect of the PAR-1 antagonist on the neuronal and nonneuronal differentiation of WT and tg cultures. BMS (1 *μ*M) significantly reduced enhanced neuronal differentiation and counteracted reduction in NG2^+^ cells in tg cultures (^*∗*^ *p* < 0.05; ANOVA).

PAR-1 is a GPCR that is activated by proteolytic cleavage within N-terminal domain. The receptor is expressed on neurons and glia in several brain regions (including hippocampus) [8, 27] and on NPC in the subventricular zone [28]. To determine whether PAR-1 is expressed in NPC preparations from murine dentate gyrus, we performed a Western blot on cell lysates, using murine hippocampus as a control. As shown in Figure 3(a), a single predominant band of the expected molecular weight size (46 kDa) was detected in both samples. To confirm that MMP activity contributes to enhanced proliferation of tg aNPCs, we tested a broad spectrum inhibitor of MMP activity (MMP inhibitor II, MMPi, 8 *μ*M) for its potential to inhibit this effect. As shown in Figure 3(b), inhibition of MMP activity significantly reduced aNPC proliferation in tg cultures at 24 h time point (^*∗*^ *p* < 0.05; ANOVA) and significantly reduced proliferation in both WT and tg cultures at 48 h (^*∗*^ *p* < 0.05; ANOVA). Fold change is shown (baseline CPS values were 71,863 ± 11,369 for WT and 77,744 ± 7898 for MMP-1 tg). We next examined a PAR-1 antagonist for its potential to inhibit enhanced proliferation of aNPCs from MMP-1 tg animals. The PAR-1 antagonist BMS-200261 (BMS), used at 1 *μ*M concentration, significantly reduced proliferation of tg-derived aNPCs, and not that of WT cultures, at 24 h and 48 h time points (Figure 3(c)). Fold change is again shown (baseline CPS values were 34,496 ± 2,971 for WT and 34,872 ± 3,962 for MMP-1 tg). In parallel, as shown in Figure 3(d), the same treatment abolished increased neuronal differentiation in tg-derived aNPC (^*∗*^ *p* < 0.05; ANOVA). Moreover, BMS counteracted the reduction in the percentage of NG2^+^ oligodendrocyte precursors observed in tg cultures (Figure 3(d)) (^*∗*^ *p* < 0.05; ANOVA). No significant effect was elicited by BMS on both neuronal and nonneuronal populations in WT-derived aNPC (Figure 3(d)). In addition, cell survival was not affected by the compound (mean percentage ± SD of viable cells: WT: 94.9 ± 1; WT + BMS: 95.6 ± 1.2; MMP-1 tg: 95.6 ± 1.4; MMP-1 tg + BMS: 95.9 ± 1.6, ANOVA).

### 3.4. The Nuclear Translocation of NF-*κ*B p65 Is Required for MMP-1 tg Associated Changes in NPC Proliferation and Neuronal Differentiation

**Figure 4:**
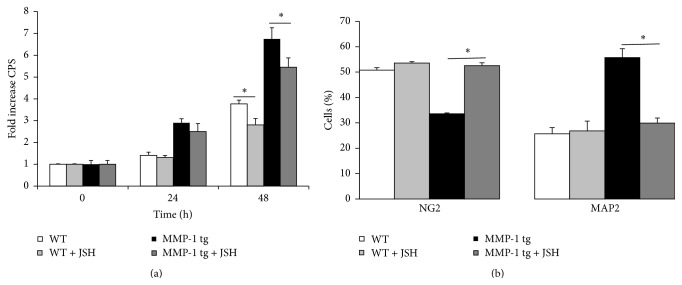
The nuclear translocation of NF-*κ*B is required for MMP-1 tg associated changes in NPC proliferation and differentiation. (a) Effect of JSH-23 on aNPC proliferation in WT and tg cultures. The drug, tested at 3 *μ*M, reduced proliferation of aNPCs in both WT and tg cultures at 48 h. (b) Effect of JSH-23 on aNPC differentiation in WT and tg cultures. 3 *μ*M JSH did counteract enhanced neuronal differentiation (^*∗*^ *p* < 0.05; ANOVA) and reduced NG2^+^ cell number (^*∗*^ *p* < 0.05; ANOVA) in tg cultures, with no effect on WT aNPC.

Previous studies have linked PAR-1 to enhanced NF-*κ*B dependent transcription of genes including VEGF. Moreover, published studies suggest that NF-*κ*B signaling is important to AHN [29–31]. We therefore tested an inhibitor of NF-*κ*B signaling for its effects on NPC proliferation and differentiation in WT and tg cultures. As shown in Figure 4(a), the inhibitor of NF-*κ*B p65 nuclear translocation JSH-23 (JSH, 3 *μ*M) reduced proliferation of aNPCs in both WT and tg cultures at 48 h. Fold change is shown (baseline CPS data values were 23,239 ± 532 for WT and 24,129 ± 4202 for MMP-1 tg). Moreover, JSH significantly reduced enhanced neuronal differentiation in tg-derived but not wt-derived cultures (^*∗*^ *p* < 0.05; ANOVA). In parallel, JSH also counteracted the reduction in the percentage of oligodendrocyte precursors in tg-derived and not wt-derived aNPC (Figure 4(b)) (^*∗*^ *p* < 0.05; ANOVA). Of note, JSH did not have a significant effect on MMP-1 levels in tg cultures (*data not shown*) and did not reduce cell survival in WT or tg cultures (mean percentage ± SD of viable cells: WT: 96.8 ± 0.97; WT + JSH: 96.5 ± 1.24; MMP-1 tg: 95.8 ± 0.7; MMP-1 tg + JSH: 95.3 ± 0.9, ANOVA).

### 3.5. SOX2 Positive NPCs Are Increased in the Hippocampus of MMP-1 tg Animals

**Figure 5:**
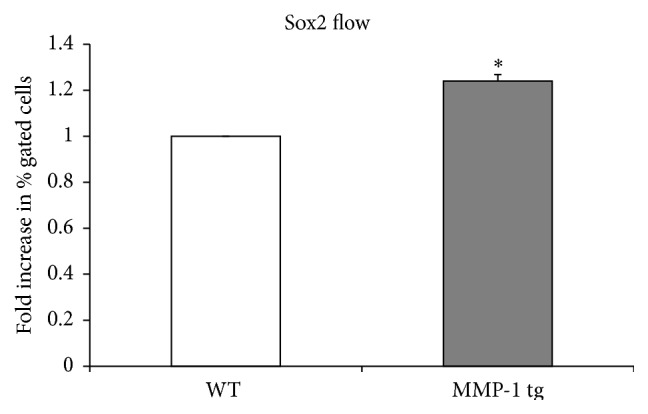
SOX2 positive NPCs are increased in the hippocampus of MMP-1 tg animals, compared to WT mice. Flow cytometric analysis of cell suspensions from murine hippocampus shows that the percentage of SOX2^+^ cells is significantly increased in tg compared to WT mice (*n* = 4 hippocampi per group with duplicate samples run in each of two separate experiments; ^*∗*^ *p* < 0.05, ANOVA).

Sequential actions of specific transcription factors are critical to proper development of AHN [32]. SOX2 is a transcription factor expressed in undifferentiated progenitor cells. It is a major mediator of Notch signaling and it is important to maintenance of the precursor pool in the adult subgranular zone (SGZ) of the DG [32–34]. We therefore examined SOX2 positive cell number in WT and MMP-1 tg hippocampus as a proxy for differences in the size of this pool. As shown in Figure 5, flow cytometric analysis of cell suspensions from the hippocampus suggests that the percentage of SOX2 positive cells is increased in MMP-1 tg compared to WT mice (*n* = 4 hippocampi per group with duplicate samples run in each of two separate experiments; ^*∗*^ *p* < 0.05).

## 4. Discussion

AHN is important to pattern separation and cognitive flexibility [2, 3]. It may also play a role in mood regulation [35]. Consistent with this possibility, cancer treatments that ablate AHN are associated with depression and anxiety [35, 36]. Moreover, AHN is necessary for serotonin reuptake inhibitors to prevent behavioural correlates of depression in animal models [37, 38].

MMPs are increasingly recognized as important effectors of neuronal plasticity in both physiological and pathological settings [39–43]. In the adult brain, these enzymes are critical to selected forms of synaptic plasticity and recent work suggests they may also contribute to AHN. For example, broad spectrum MMPi reduces proliferation and neuronal differentiation of neural stem cells from umbilical cord blood [44], and MMP activity contributes to stroke-induced neurogenesis [45]. Moreover, MMP-2 expression is detected in SOX2 immunopositive progenitor cells in the teleost fish, while siRNA to select MMPs abrogates enhanced neurogenesis in a murine model of intracerebral haemorrhage [46, 47]. Unanswered questions include those related to whether specific MMPs are sufficient to drive enhanced neurogenesis and those related to the mechanisms through which MMPs can enhance aNPC proliferation and/or differentiation.

In the present study, we show that overexpression of MMP-1 is sufficient to drive increased proliferation of aNPCs* in vitro*. Moreover, these data were corroborated by flow cytometry data demonstrating the presence of a significantly higher number of SOX2 positive cells in hippocampi of MMP-1 tg mice when compared to their WT counterpart.* In vitro*, MMP-1 overexpression also stimulates enhanced neuronal fate specification of progenitors, with no changes in astrocyte generation and a parallel reduction in the number of NG2^+^ oligodendrocyte precursors. Importantly, increased proliferation and enhanced neuronal differentiation of MMP-1 tg-derived aNPC could be abrogated by an inhibitor of PAR-1 signaling. As compared to the broad spectrum MMPi, which reduced proliferation of both WT and tg aNPCs, a PAR-1 antagonist did not substantially influence this endpoint in WT cultures. Differential effects of MMP versus PAR antagonists could relate to release of murine-encoded MMPs from tg cultures that act through PAR-1 independent mechanisms including proneurotrophin conversion [48]. Broad spectrum inhibition of MMP activity may thus influence neurogenesis at many levels and differences in both cell proliferation and survival should be considered.

We also show that an inhibitor of NF-*κ*B p65 nuclear translocation, JSH-23, reduces neuronal differentiation in a MMP-1 tg selective manner. This is consistent with prior work in which JSH-23 did not diminish neuronal differentiation when administered in isolation but did diminish enhanced neuronal differentiation in response to a drug that could activate the NF-*κ*B pathway and in parallel increased hippocampal neurogenesis both* in vitro* and* in vivo* [31]. Though additional studies will be necessary to determine whether MMP-1 is a relatively direct effector of NF-*κ*B signaling, data herein are consistent with the possibility that NF-*κ*B activation is increased in tg-derived aNPCs.

PAR-1 stimulated effects on aNPC proliferation and differentiation are not without precedent. Previous studies with PAR-1 agonists that may enter the CNS with injury, including activated protein C (APC) and thrombin [49–51], suggest this receptor may be important to AHN which follows ischemic or hemorrhagic insult. APC analogues can also promote neurogenesis in rodents following focal ischemic stroke [52]. Effects of APC and thrombin on hippocampal neurogenesis may, however, be most relevant to select pathological states in which blood brain barrier damage occurs. Moreover, as compared to thrombin and APC, MMP-1 generates a unique peptide agonist with the potential to stimulate distinct intracellular signaling events [12]. Thrombin, APC, and MMP-1 cleave PAR-1 at arginine 41, arginine 41 or 46, and aspartate 39, respectively. The MMP-1 generated tethered ligand is thus longer by at least two amino acids. This is important in that small differences in GPCR agonist structure can alter receptor localization, G protein coupling, and the duration of signaling [53]. Consistent with this, Blackburn and Brinckerhoff have shown that equimolar concentrations (5 nM) of MMP-1 and thrombin have differential effects on human microvessel endothelial cells. MMP-1 stimulates relatively long-lived increases in p38 and MEK/ERK signaling and gene expression analysis also showed differences as a function of agonist [12]. Intriguingly, MMP-1 stimulated a 3-fold increase in the expression of the proneurogenic molecule VEGFA, while there was no increase of VEGFA expression in response to thrombin [12, 54]. Additional work has linked PAR-1 signaling to enhanced release of MMP-9 [55], an additional potential effector of neuronal differentiation [45].

With respect to future studies and the question of the MMP-1/PAR-1 axis in regulating AHN under physiological and/or pathological conditions, it should be noted that MMP-1 expression may be increased in association with select physiological and pathological stimuli relevant to neurogenesis. Future studies will be necessary to determine whether PAR-1 activating MMPs are important for neurogenesis in the background of environmental enrichment and/or antidepressant medication. MMP-1 levels may also be increased in disorders of the central nervous system characterized by glial activation. Such disorders include fragile X syndrome [56], a condition in which enhanced neurogenesis may be observed [57]. Given that the MMP-1 promoter is driven by transcription factors including AP-1, which are strongly modulated by excitatory neurotransmission [58], future studies might also address MMP-1 dependent neurogenesis as a function of seizure activity or electroconvulsive therapy.

## 5. Conclusions

Herein we suggest that the MMP-1/PAR-1 axis can significantly impact both the proliferation and differentiation of aNPCs derived from hippocampus. Additional experiments may be warranted to determine whether this axis contributes to neurogenesis-related behaviors including cognitive flexibility and responsiveness to antidepressant therapy. Future studies might also examine the relevance of this axis to postinjury neurogenesis.
